# Cranial MRI beyond the Neonatal Period and Neurodevelopmental Outcomes in Neonatal Encephalopathy Due to Perinatal Asphyxia: A Systematic Review

**DOI:** 10.3390/jcm12247526

**Published:** 2023-12-06

**Authors:** Corline E. J. Parmentier, Tobias Kropman, Floris Groenendaal, Maarten H. Lequin, Linda S. de Vries, Manon J. N. L. Benders, Thomas Alderliesten

**Affiliations:** 1 Department of Neonatology, Wilhelmina Children’s Hospital and Utrecht Brain Center, University Medical Center Utrecht and Utrecht University, 3584 EA Utrecht, The Netherlands; 2 Department of Radiology, Wilhelmina Children’s Hospital and Utrecht Brain Center, University Medical Center Utrecht and Utrecht University, 3584 EA Utrecht, The Netherlands

**Keywords:** neuro-imaging, perinatal asphyxia, neonatal encephalopathy

## Abstract

Background: Magnetic resonance imaging (MRI) including diffusion-weighted imaging within seven days after birth is widely used to obtain prognostic information in neonatal encephalopathy (NE) following perinatal asphyxia. Later MRI could be useful for infants without a neonatal MRI or in the case of clinical concerns during follow-up. Therefore, this review evaluates the association between cranial MRI beyond the neonatal period and neurodevelopmental outcomes following NE. Methods: A systematic literature search was performed using PubMed and Embase on cranial MRI between 2 and 24 months after birth and neurodevelopmental outcomes following NE due to perinatal asphyxia. Two independent researchers performed the study selection and risk of bias analysis. Results were separately described for MRI before and after 18 months. Results: Twelve studies were included (high-quality *n* = 2, moderate-quality *n* = 6, low-quality *n* = 4). All reported on MRI at 2–18 months: seven studies demonstrated a significant association between the pattern and/or severity of injury and overall neurodevelopmental outcomes and three showed a significant association with motor outcome. There were insufficient data on non-motor outcomes and the association between MRI at 18–24 months and neurodevelopmental outcomes. Conclusions: Cranial MRI performed between 2 and 18 months after birth is associated with neurodevelopmental outcomes in NE following perinatal asphyxia. However, more data on the association with non-motor outcomes are needed.

## 1. Introduction

Neonatal encephalopathy (NE) following perinatal asphyxia is a major cause of neonatal death and impaired neurodevelopment [1]. Therapeutic hypothermia started within six hours after birth is currently the only neuroprotective intervention that is proven to reduce death and adverse neurodevelopmental outcomes among (near-)term infants with moderate and severe NE [2]. However, a significant number of infants still suffer from brain injury and subsequent adverse neurodevelopmental outcomes despite therapeutic hypothermia, including cerebral palsy (CP), epilepsy, and behavioral or cognitive impairments [3,4]. 

Cranial magnetic resonance imaging (MRI), including diffusion-weighted imaging (DWI), and proton magnetic resonance spectroscopy are currently the gold standard to assess the degree of brain injury following perinatal asphyxia and to gain prognostic information to counsel families and guide further treatment decisions in the neonatal period [5]. Neonatal MRI is preferably performed within one week after birth, before pseudo-normalization of acute injury visible on DWI occurs [6]. Several studies have demonstrated that neonatal MRI is associated with neurodevelopmental outcome [6,7,8]. However, its predictive value is dependent on the timing of imaging, the quality of the scans, and the scoring system that is being used to quantify the degree of injury [5]. Acute profound asphyxia is associated with deep gray matter (DGM) injury, which is known to be related to impaired motor neurodevelopment. Partial prolonged asphyxia often results in white matter and/or watershed injury, which has been related to later cognitive problems [5,9].

MRI beyond the neonatal period could be considered to assess the evolvement of injury and residual damage in infants with abnormalities on the neonatal scan, to assess injury in infants who could not be scanned within the optimal time frame for DWI, or in the case of ongoing clinical concerns during follow-up [10]. Furthermore, a repeat MRI could be used as an early biomarker in neuroprotective trials before long-term follow-up data become available. The sequelae of brain injury due to perinatal asphyxia evolve over time, and may include gliosis, atrophy, cyst formation, and/or delayed myelination at later imaging [10,11]. Importantly, as myelination continues in the second year after birth, an MRI obtained during infancy may not yet demonstrate the full extent of brain injury following perinatal asphyxia [12].

Although the association between neonatal MRI and neurodevelopmental outcomes following perinatal asphyxia has been well-studied, little is known about the prognostic value of MRI performed beyond the neonatal period. Therefore, the aim of this systematic review was to determine the association between cranial MRI in the initial months following the neonatal period (2–18 months after birth) and near completion of myelination (18–24 months after birth) and neurodevelopmental outcomes following NE due to perinatal asphyxia. 

## 2. Materials and Methods

### 2.1. Information Sources and Search Strategy 

This systematic review was conducted according to the Preferred Reporting Items for Systematic Reviews and Meta-Analyses guidelines and registered in PROSPERO (no. CRD42022351343). PubMed and Embase were searched for studies on cranial MRI performed between 2 and 24 months after birth in relation to neurodevelopmental outcome at ≥3 months of age in infants with NE following perinatal asphyxia. There were no restrictions on the publication date, and the last update of the search was on 4 May 2023. An overview of the search strategy including the entry terms is demonstrated in Appendix A.

### 2.2. Study Selection

Two reviewers (CEJP and TK) independently performed title and abstract screening using the Rayyan application [13]. In the case of a disagreement, a third reviewer (TA) was consulted. The inclusion criteria were: (1) infants with NE following perinatal asphyxia, as defined by every individual study, (2) cranial MRI performed between 2 and 24 months of age, (3) neurodevelopmental outcome ≥3 months of age reported in relation to later MRI, and (4) articles written in English. Articles describing only preterm infants, animal studies, conference papers, reviews, and case reports were excluded. Subsequently, the full text of the articles selected based on title and abstract screening were assessed by two independent reviewers (CEJP and TK). A third reviewer (TA) was consulted in the case of a disagreement.

### 2.3. Data Extraction and Quality Assessment

Data from the selected studies were extracted using an adjusted data extraction form from the CHARMS-PF guidelines [14]. In the case of missing information, the authors were contacted to request the data. For articles in which statistical analysis was not performed or reported, the data that were described by the study were analyzed using SPSS statistics 29.0 (IBM Inc., Chicago, IL, USA). The included studies were critically appraised by two independent researchers (CEJP and TK) using an adjusted version of the quality in prognosis studies (QUIPS) review form (Appendix B and Supplement S1). Twenty-six items on study participation, study attrition, prognostic factor measurement, outcome measurement, confounding, and statistical analysis and reporting were comprehensively reviewed in terms of methodological quality. Consensus was reached by discussion, and in the case of a disagreement, a third reviewer (TA) was consulted. Studies were classified as high-quality when at least three domains were graded as low risk without any domains graded as high risk of bias, and as low-quality when at least three domains were graded as high risk of bias. The remaining articles were graded as medium-quality. 

### 2.4. Synthesis of Results 

Due to the heterogeneity in studies for the study population, definition of later MRI, and assessment of neurodevelopmental outcome, a meta-analysis could not be performed. Therefore, a best-evidence synthesis was applied using the following three levels: (1) strong evidence: consistent findings in ≥2 high-quality studies; (2) moderate evidence: consistent findings in 1 high-quality and at least 1 moderate- or low-quality study, or consistent findings in ≥2 moderate- or ≥3 low-quality studies; and (3) insufficient evidence: only 1 study available or inconsistent findings in multiple studies [15]. As the sequelae of neonatal brain injury evolve over time, and myelination is best appreciated on MRI performed in the second year after birth, the results were separately described for MRI performed at 2–18 months after birth and MRI performed at 18–24 months after birth. 

## 3. Results

### 3.1. Article Search

**Figure 1:**
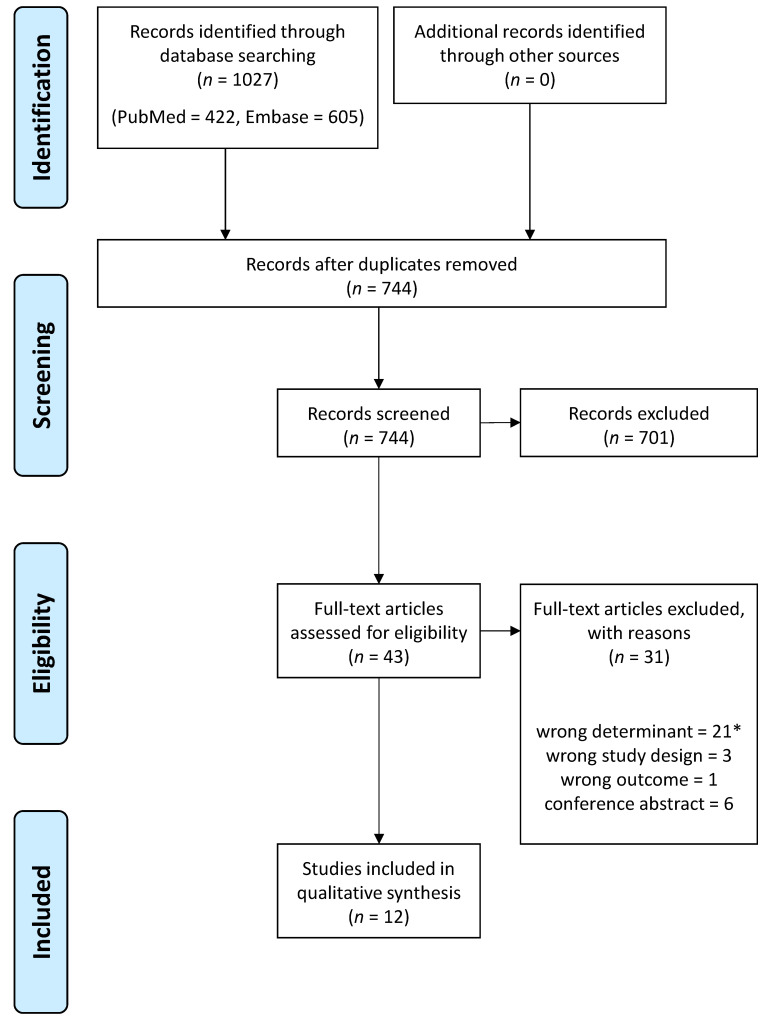
Flow diagram of the study selection. * One article was excluded because later MRI was performed beyond 24 months after birth according to information from the authors.

A total of 1027 records were collected from PubMed and Embase. After the removal of duplicates, 744 unique records were screened by title and abstract. Forty-three articles were eligible based on the inclusion and exclusion criteria. Thirteen articles were included after full-text screening. One article was subsequently excluded because later MRI was performed beyond 24 months after birth according to information from the authors. Finally, 12 articles were included (Figure 1). Two studies were rated as high-quality, six were rated as moderate-quality, and four were rated as low-quality (Table A1). 

### 3.2. Study Characteristics

The characteristics of the included studies are presented in Table 1. Three articles were published within the past ten years. Most studies included only infants with gestational age ≥36.0 weeks (*n* = 8, 67%). In three studies, only a subset of the study population had a history of perinatal asphyxia [16,17,18]. Age at last follow-up assessment ranged from 3 months to 17 years of age. Most studies used the Griffiths Mental Development Scales (*n* = 4) and/or different editions of the Denver Developmental Screening Test (*n* = 4) and the Bayley Scales of Infant and Toddler Development (*n* = 3) to assess neurodevelopmental outcomes. All twelve studies analyzed infants with a later MRI performed within 18 months after birth [10,16,17,18,19,20,21,22,23,24,25,26] and three studies also described a subset of infants with MRI performed at 18–24 months after birth [10,16,24]. For six papers, the data as presented by the study were re-analyzed in SPSS, as a complete statistical analysis was not provided in the article.

### 3.3. MRI at 2–18 Months

#### 3.3.1. Composite Adverse Outcomes

The association between MRI at 2–18 months and neurodevelopmental outcomes as defined by each individual study is summarized in Table 2. Seven studies (high-quality *n* = 2, moderate-quality *n* = 2, low-quality *n* = 3) described the association between qualitative findings on MRI performed at 2–18 months and a composite neurodevelopmental outcome. All demonstrated a significant association between later MRI abnormalities and the composite adverse outcome as defined by the specific study [10,17,18,19,22,23,26]. Three studies, one graded as high-quality and two as moderate-quality, demonstrated a significant association between biometrics on MRI performed at 2–18 months and a composite neurodevelopmental outcome [10,20,21]. Parmentier et al. and Spring in ‘t Veld et al. demonstrated that 1D and 2D biometrics of the DGM on 3-month MRI were associated with adverse neurodevelopmental outcomes [10,20]. Noteworthy, 24 of the 63 infants from the study by Parmentier et al. were also included in the study by Spring in ‘t Veld and colleagues. Mulkey et al. reported that whole brain volumes on MRI performed at ≥2 months were associated with adverse neurodevelopmental outcomes, which was determined at a median age of 556 (range 255–1556) days [21].

#### 3.3.2. Motor Outcomes

Cranial MRI findings at 2–18 months in relation to motor outcome were described in six studies, of which three moderate-quality studies demonstrated a significant association [16,21,22,23,24,25]. Byrne and colleagues demonstrated a significant association between 8-month MRI abnormalities, including structural abnormalities, delayed myelination, and/or dilated ventricles, and CP at 18 months of age [25]. Belet et al. showed that the presence of encephalomalacia, atrophy, DGM injury, and/or white matter lesions on 4-month MRIs was significantly associated with CP at 4 years of age [22]. Analysis of the data presented by Tekgul et al. demonstrated that among infants with abnormal MRI between 4 and 12 months, those with only delayed myelination were less likely to show a motor deficit at two years of age compared with the other brain injury patterns [23]. In three studies, a significant association between MRI findings at 2–18 months and motor outcomes could not be demonstrated: Mulkey and colleagues reported that infants with decreased whole brain volumes on follow-up MRI had higher odds of demonstrating the combination of CP, epilepsy, and neurodevelopmental delay but did not show an association with motor impairment as a separate outcome [21]. Subgroup analysis of 13 infants with a history of perinatal asphyxia and MRI performed at 2–18 months described by Steinlin et al. did not show a significant association between diffuse brain MRI injury (demonstrated in *n* = 6) and motor abnormalities at follow-up [24]. Krägeloh-Mann and colleagues described three infants with a history of NE due to perinatal asphyxia with a 12-month MRI, but a statistical analysis to demonstrate an association between MRI findings and motor outcomes was not possible for this small group [16]. 

Four articles reported on the pattern of injury on later MRI in relation to motor outcome following asphyxia [22,23,24,26]. The most commonly described patterns of injury in infants with adverse motor outcomes were focal or diffuse cystic encephalomalacia, periventricular signal intensity changes or periventricular leukomalacia (PVL), white matter atrophy, delayed myelination, and DGM injury. Tekgul et al. showed that among infants with abnormal MRI at 4–12 months, those with only focal cortical involvement or myelination delay were most likely to have a favorable outcome in contrast to infants with PVL, marked cortical atrophy, DGM injury, or multi-cystic encephalomalacia [23]. 

#### 3.3.3. Cognitive Outcomes

Two articles described cognitive abnormalities in relation to MRI findings at 2–18 months [16,18]. Millet et al. analyzed 60 infants with a perinatal neurologic insult, including 15 term infants with perinatal asphyxia. For the whole group, diffuse brain injury, which was not further specified, on later MRI was significantly associated with cognitive problems at 2–5 years of age. No data on cognitive impairments were reported for the subgroup of term infants with asphyxia [18]. Krägeloh-Mann and colleagues reported that the three infants with a history of perinatal asphyxia, who demonstrated mild (*n* = 1) and severe DGM lesions (*n* = 2) on 12-month MRI, had a mild cognitive delay (assessed at 15 months) and a severe cognitive delay at, respectively, 27 and 99 months [16].

#### 3.3.4. Epilepsy 

Five studies explored brain MRI findings at 2–18 months in relation to the development of epilepsy [18,21,22,23,24]. In two papers, a statistical analysis to demonstrate an association between MRI findings and epilepsy was not possible due to limited sample sizes [18,24]. Three had sufficient data for a statistical analysis. Among these, one moderate-quality study demonstrated a significant association: analysis of the data presented by Belet et al. showed that both 4-month (*p* = 0.033) and 4-year MRI abnormalities (*p* = 0.012) were associated with epilepsy. Patterns of injury on 4-month MRI among the eight infants who developed epilepsy were focal encephalomalacia with (*n* = 3) or without atrophy (*n* = 1), DGM injury (*n* = 1), PVL (*n* = 1), and diffuse cystic encephalomalacia with atrophy (*n* = 1). The remaining infant had a normal 4-month MRI. On 4-year MRI, these infants showed focal encephalomalacia with (*n* = 3) or without atrophy (*n* = 1), focal encephalomalacia with PVL (*n* = 1), and DGM injury (*n* = 1). The other two infants had no 4-year MRI performed. Two studies did not show a significant association between MRI findings and epilepsy: Mulkey and colleagues reported that acute injury volume in the corpus callosum on neonatal MRI was significantly associated with the later development of epilepsy but did not report an association between 2–18-month MRI findings and epilepsy as a separate outcome [21]. Tekgul and colleagues described 24 infants who developed epilepsy among their study population of 65 term asphyxiated infants with abnormal MRI at 4–12 months. They showed PVL (*n* = 7), marked cortical atrophy (*n* = 6), multicystic encephalomalacia (*n* = 6), DGM injury (*n* = 3), and focal cortical involvement (*n* = 2), but a significant association with one of these injury patterns could not be demonstrated. 

#### 3.3.5. Audiovisual Impairment 

Cranial MRI at 2–18 months in relation to hearing and visual impairment was described by *n* = 2 [18,24] and *n* = 3 articles [18,23,24], respectively. The number of infants with audiovisual impairments was too small to perform a statistical analysis to explore the association with MRI findings. Millet et al. described two infants with sensorineural hearing loss among their cohort of term and preterm infants considered at risk of neurologic impairment. Both showed diffuse brain damage on MRI performed at 3–6 months corrected age. Fourteen infants developed a visual impairment (strabismus *n* = 12, cortical blindness *n* = 1, unspecified visual impairment *n* = 1): twelve showed diffuse brain damage on later MRI, and two showed focal brain damage, without details on the location. Whether these infants had a history of perinatal asphyxia was not described [18]. Steinlin and colleagues described one infant with hearing loss, showing severe cerebral necrosis and a thin corpus callosum on a 10-month MRI. Four infants had visual impairment (blindness *n* = 3, visual retardation not further specified *n* = 1); all infants who developed blindness had diffuse cerebral necrosis on MRI at 2–18 months, and the infant with delayed visual development showed severe frontal atrophy [24]. Tekgul et al. reported on seven infants with unspecified visual impairments, who showed multicystic encephalomalacia (*n* = 4) and PVL (*n* = 3) on MRI performed at 4–12 months [23].

### 3.4. MRI at 18–24 Months

Three papers described infants with later MRI performed at 18–24 months of age. A statistical analysis to demonstrate an association with neurodevelopmental outcomes was not possible due to the small number of infants. In the study by Krägeloh-Mann and colleagues, two infants with a history of perinatal asphyxia had MRI performed at 18–24 months: both had mild DGM injury and developed mild CP. Their cognitive abilities at, respectively, 105 and 75 months of age were normal [16]. Steinlin et al. described two infants with MRI at 18 and 19 months after birth, respectively: one showed severe cerebral necrosis and a thin corpus callosum and had severe quadriplegia, blindness, and microcephaly at 24 months of age. The other demonstrated minimal frontal atrophy on MRI and had mild quadriplegia and a normal developmental quotient at 18 months of age [24]. Parmentier et al. described a subset of 12 infants who had MRI performed at both three months and 18–24 months of age to assess the evolution of injury in infants with (suspected) CP, extensive neonatal MRI injury, or abnormalities on neurological examination not explained by previous neuroimaging. All infants showed more extensive abnormal WM signal intensity, suggestive of gliosis compared with 3-month MRI. At 5.5 years of age, all had an adverse outcome: five had an adverse cognitive outcome, six had an adverse motor outcome, and one had both an adverse cognitive and motor outcome [10]. 

### 3.5. Neonatal MRI versus Later MRI

Eight studies analyzed both neonatal and later MRI in relation to neurodevelopmental outcomes (Table 3). Three studies (moderate-quality *n* = 1, low-quality *n* = 2), concluded that later MRI findings correlated better with neurodevelopmental outcome than conventional neonatal MRI without DWI [17,24,25]. Two articles, one of high quality and one of moderate quality, reported that neonatal MRI had better sensitivity and negative predictive value, whereas later MRI had better specificity and positive predictive value for an adverse neurodevelopmental outcome [10,22]. In one of these studies, DWI was part of the neonatal MRI protocol [10]. The remaining studies all reported an association between neonatal MRI findings and neurodevelopmental outcomes but did not draw further conclusions on whether neonatal MRI was preferable over later MRI.

### 3.6. Therapeutic Hypothermia

Three studies (high-quality *n* = 1, moderate-quality *n* = 2) included infants who received therapeutic hypothermia. All 16 infants included by Mulkey et al. received hypothermia [21]. Infants with whole brain volumes below the lower limit of the 99% confidence interval of the volume estimation model for MRI performed ≥ 2 months had increased odds of developing epilepsy, CP, and neurodevelopmental delay (odds ratio: 33, 95% CI: 2.32–469.03, *p* = 0.008), whereas infants with whole brain volumes above the upper limit of the 99% confidence interval had an odds ratio of 15 for a normal outcome (95% CI: 1.21–185.46, *p* = 0.029). Parmentier et al. included 28 infants who received hypothermia and showed in a subgroup analysis that cooled infants with an adverse 18–24-month outcome had a larger frontal horn depth (*p* = 0.015) and smaller DGM surface area (*p* = 0.005) on 3-month MRI [10]. They demonstrated higher total injury scores and DGM sub-scores for 3-month MRI than cooled infants with a normal 18–24-month outcome. Spring in ‘t Veld et al. included three infants treated with therapeutic hypothermia but did not describe these infants separately [20].

## 4. Discussion

In this systematic review, the association between cranial MRI performed beyond the neonatal period and neurodevelopmental outcomes in infants with NE following perinatal asphyxia was evaluated. There was considerable heterogeneity concerning the timing and assessment of later MRI, the measurement of outcomes, and the duration of follow-up. Based on the findings of two high-quality, two moderate-quality, and three low-quality studies, there is strong evidence for an association between qualitative abnormalities on MRI performed between 2 and 18 months of age and an overall adverse neurodevelopmental outcome, with both the location and the severity of brain injury being associated with adverse neurodevelopment. According to the findings of three moderate-quality studies, there is moderate evidence for an association between abnormalities on MRI performed at 2–18 months and impaired motor development. Based on one high-quality and one moderate-quality study, there is moderate evidence for an association between 2–18-month MRI abnormalities and adverse neurodevelopmental outcomes among infants who received therapeutic hypothermia. There were insufficient data on the association between MRI performed between 18 and 24 months and neurodevelopmental outcomes and on the association between later MRI and non-motor outcomes. 

Currently, neonatal MRI with DWI performed as soon as possible after therapeutic hypothermia is widely recommended to assess the degree of brain injury and obtain prognostic information in NE following perinatal asphyxia [5]. Early MRI has a good predictive value for long-term outcomes, especially since the implementation of DWI and magnetic resonance spectroscopy [5]. However, performing MRI within the optimal time frame of DWI (i.e., before pseudo-normalization of injury at 8–10 days after the insult) is not always possible, for instance, when infants are too ill to undergo MRI. Furthermore, infants may not develop as expected based on the findings from early MRI. 

The results of this review indicate that MRI beyond the neonatal period could be useful in these newborns, in particular, to gain information on motor impairments. However, there are insufficient data from the papers in this review to determine the optimal timing when MRI beyond the neonatal period is considered valuable. Brain injury due to perinatal asphyxia evolves over time, resulting in gliosis, atrophy, cyst formation, and/or delayed myelination [10,11]. As myelination completes after the second year after birth, the presence of gliosis and impaired myelination might not be visible to its full extent when MRI is repeated too early [12]. Only four papers included infants in whom later MRI was performed between 18 and 24 months after birth. Based on our own experience, we previously described that in a subgroup of infants for whom MRI was performed at both 3 months and 18–24 months after birth, all showed more extensive white matter gliosis on MRI at 18–24 months compared with the 3-month MRI [10]. However, MRI in this subgroup was performed at 18–24 months because of (suspected) CP, extensive neonatal MRI injury, or neurologic abnormalities not explained by prior MRI. These findings are therefore not generalizable to the overall group of infants with NE due to perinatal asphyxia. Another study, in which myelination was only assessed at 4 years, demonstrated better sensitivity and negative predictive value of 4-year MRI compared with 4-month MRI [22]. Although it may be postulated that MRI beyond 18 months after birth would be most informative, the need for early information for the timely provision of developmental support and the difficulty of scanning young children should be taken into account [31].

The main reported patterns of injury on later MRI that were associated with adverse motor outcomes included cystic encephalomalacia, periventricular signal intensity changes or PVL, white matter atrophy, delayed myelination, and DGM injury. Unfortunately, the patterns of injury in infants with abnormal cognitive outcomes were underreported. For neonatal MRI, in particular, watershed injury has been demonstrated to be correlated to cognitive impairments [32,33]. Therefore, it can be hypothesized that the sequelae of neonatal white matter and watershed injury on later MRI (e.g., cyst formation and gliosis) could precede later cognitive problems. Recently, it was demonstrated that injury to the hippocampi and mammillary bodies on MRI at 10 years was associated with neurocognition and memory at school age in children with a history of NE due to perinatal asphyxia, suggesting that atrophy of the hippocampi and mammillary bodies could also be an early biomarker for neurocognitive and memory problems [4].

Therapeutic hypothermia is currently the only neuroprotective intervention to minimize death and adverse neurodevelopmental outcomes among (near-)term infants with moderate–severe NE following perinatal asphyxia [2]. However, a substantial number of infants still have an adverse neurodevelopmental outcome despite cooling or do not qualify for hypothermia [3,34]. Several other neuroprotective strategies are currently being investigated to improve the outcome of infants with NE [35]. The results from this review suggest that later MRI could be an appropriate biomarker in research on additive therapies for therapeutic hypothermia, as well as studies on new strategies for infants who are currently not eligible for cooling. Unfortunately, the number of available studies reporting on non-motor outcomes was limited. Furthermore, only two studies assessed neurodevelopmental outcomes beyond five years of age [11,16]. There is increasing evidence that especially cognitive and behavioral problems following perinatal asphyxia may become apparent at a later age [3,4]. The question remains as to whether later MRI is also associated with long-term non-motor outcomes, which could not be determined from the data in this review.

The limitations of this review include the high variability among the studies regarding the timing and methods for later MRI, as well as the duration of follow-up and the assessment of neurodevelopmental outcomes. Different definitions for perinatal asphyxia and NE were used, and some studies also included preterm infants and infants with neonatal brain injury due to other causes than NE, without reporting data on the subgroup of term infants with perinatal asphyxia. A meta-analysis was therefore not possible. Secondly, more than half of the studies were performed over twenty years ago, limiting the generalizability of our results to current clinical practice. Thirdly, data on possible confounders were often not reported, and most studies did not perform a sufficient statistical analysis to correct for these factors. Fourthly, there were limited data on infants who received therapeutic hypothermia and the association between later MRI and non-motor outcomes. Finally, it should be noted that the infants described in this review represent a selected population, as those who were most severely affected by NE did not survive and those who were only mildly affected were less likely to be scanned. 

Future studies should investigate the association between later MRI and long-term neurodevelopmental sequelae, including separate analyses of cognitive and behavioral outcomes, and the optimal timing to perform later MRI. Furthermore, advanced imaging techniques, such as diffusion-tensor imaging, could improve the predictive value of MRI beyond the neonatal period [36]. 

## 5. Conclusions

In conclusion, both qualitative and quantitative abnormalities on cranial MRI performed at 2–18 months after birth in infants with NE following perinatal asphyxia are associated with an adverse neurodevelopmental outcome, in particular, impaired motor development. Although data on infants treated with therapeutic hypothermia are limited, this association seems to be independent of the use of therapeutic hypothermia. Later MRI can be used for prognostication when a neonatal MRI could not be performed or was not performed within the optimal time frame of DWI. Furthermore, it can provide information when an infant does not develop as expected based on early MRI findings. In addition, it might serve as a biomarker in research on new neuroprotective strategies. Future studies should explore the association between later MRI findings and long-term development, including non-motor outcomes, the optimal timing to perform MRI beyond the neonatal period, and the utility of advanced imaging techniques to improve its predictive value. 

## Figures and Tables

**Table 1:** Characteristics of the included studies. ^1^: Only infants with abnormal MRI are included. ^2:^ All infants scanned <18 months after birth; ^3^: MRI findings were only reported for 24 infants, of whom 21 had MRI > 1 month. ADHD: attention deficit hyperactivity disorder; Bayley-III-NL: Dutch version of the Bayley Scales of Infant and Toddler Development—Third Edition; CA: corrected age; CP: cerebral palsy; DDST: Denver Developmental Screening Tests; GA: gestational age; GMDS: Griffiths Mental Development Scales; mo: months; no: number; P: prospective; PA: perinatal asphyxia; R: retrospective; U: unknown; WPPSI-III-NL: Dutch version of the Wechsler Preschool and Primary Scale of Intelligence—Third edition; y: year.

Author, Year of Publication	Design	Total No./No. Term PA	GA (w)	Age at Later MRI	Age at Follow-Up	Neurodevelopmental Test	Definition of (Composite) Adverse Outcome	Study Quality
Parmentier, 2023 [10]	R	63/63	≥36.0	2–4 mo (all) 18–24 mo (subset)	18–24 mo (all) 5.5 y (subset)	18–24 mo: Bayley-III-NL or GMDS 5.5 y: M-ABC, WPPSI-III-NL	CP, epilepsy, hearing/visual impairment, or neurodevelopmental delay	H
Jose, 2013 [19]	P	31/31	≥37.0	10–12 weeks	12 mo	Neurological examination, DDST II	Abnormal neurological exam and/or untestable DDST II	H
Spring in ‘t Veld, 2016 [20]	R	29/29	≥36.0	3 mo	≥18 mo	GMDS	CP or neurodevelopmental delay	M
Mulkey, 2012 [21]	R	16/16	≥36.0	2–18 mo	Median 556 (range 255–1556) days	Neurological examination	Epilepsy, CP, and neurodevelopmental delay	M
Belet, 2004 [22]	P	24/24	>37.0	4 mo 3.5–4 years	3.5–4 y	Neurological examination, Bayley Scales, and DDST (edition not described)	CP, developmental delay, and epilepsy	M
Tekgul, 2003 [23]	R	65/65 ^1^	≥37.0	4–12 mo	2 y	Neurological examination, DDST II	Moderate–severe neurologic abnormalities (causing functional impairment or requiring full-time special assistance) or difficult-to-control post-neonatal seizures	M
Kragelöh-Mann, 2002 [16]	R	17/9	≥34.0	≥12 mo Subset with PA: 12–18 mo *n* = 3 18–24 mo *n* = 2 >24 mo *n* = 4	18 mo–17 y	CP-recorded according to Krägeloh-Mann et al. [27] motor and cognitive development based on milestones by Largo et al. [28,29], and school results if available	Mild motor impairment: walk independently between 18 mo and 5 y, or sit between 10 mo and 2 y; severe motor impairment: unable to walk at 5 y, unable to sit at 2 y, or no head control at 1 y Mild cognitive impairment: developmental milestones < 10th centile and delay < 1 y; severe cognitive impairment: delay > 1 y or not following with eyes/establishing visual contact at age 1 y	M
Byrne, 1990 [25]	P	15/15	≥37.0	4 mo 8 mo	18 mo	Neurologic examination of the Collaborative Perinatal Project	CP	M
Millet, 1998 [18]	P	60/15	≥25.0	3–6 mo CA	2–5 y	Neurological examination with Amiel–Tison assessment, Bayley Scales, parental interview, and full ophthalmologic and audiologic assessment if screening was abnormal	CP, cognitive impairment (dysphasia/delayed expressive language, ADHD, and lack of visuospatial processing or spatialization), visual disability (strabismus or blindness), developmental delay, and seizures	L
Rutherford, 1996 [26]	U	16/16	≥37.0	12–24 mo ^2^	12–24 mo	Neurological examination and GMDS	Signs of central motor deficit with or without developmental delay	L
Fujii, 1993 [17]	U	39/U	≥24.0	2–12 mo CA	18 mo CA	Japanese Edition of DDST and Enjoji Developmental Scale	Neurodevelopmental delay and/or CP	L
Steinlin, 1991 [24]	U	30/30 ^3^	≥28.0	1–2 mo *n* = 1 2–18 mo *n* = 13 18–24 mo *n* = 2 >24 mo *n* = 5	3–6 mo (*n* = 7) 6–12 mo (*n* = 8) 2 y (*n* = 9) 2–3 y (*n* = 1)	Neurological examination, GMDS, and Snijders–Oomen nonverbal intelligence test	No definition for adverse outcome. Outcome was described for each infant, including (signs of) CP, neurodevelopmental delay, visual abnormalities, and epilepsy	L

**Table 2:** Summary of 2–18-month MRI findings and neurodevelopmental outcomes. BG: basal ganglia; CI: confidence interval; CP: cerebral palsy; DGM: deep gray matter; DWI: diffusion-weighted imaging; FLAIR: fluid attenuated inversion recovery IR: inversion recovery; NA: not available; NPV: negative predictive value; OR: odds ratio; PD: proton density; PPV: positive predictive value; SWI: susceptibility-weighted imaging; WM: white matter. * Data were analyzed in SPSS Statistics as a statistical analysis was not (fully) provided in the article.

Author, Year of Publication	Later MRI Strength, Sequences	Later MRI Assessment	Association between Later MRI and Neurodevelopmental Outcomes
Parmentier, 2023 [10]	1.5 (*n* = 27) or 3.0 T (*n* = 36); T1, T2	Biometrics: 1D and 2D measurements Qualitative: new injury score including WM, DGM and cerebellum sub-score	Biometrics: smaller DGM surface area (*p* < 0.001) and frontal horn depth (*p* < 0.001) on 3-month MRI associated with composite adverse outcomes at 18–24 months; smaller brain width (*p* = 0.027) associated with composite adverse outcomes at 5.5 years. Qualitative score: WM and DGM sub-scores for 3-month MRI associated with adverse 18–24 month outcomes. Infants with adverse outcomes at 5.5 years had higher DGM sub-scores for later MRI (*p* = 0.042). Sensitivity, specificity, PPV, and NPV of the 3-month MRI scoring model for composite adverse outcomes at 18–24 months were 65%, 95%, 88%, and 83%.
Jose, 2013 [19]	1.5 T; T1, T2, DWI, SWI	Qualitative: categorization of injury according to Barkovich [30]	Normal later MRI associated with normal outcomes (*p* < 0.001); abnormal signal in cortex and basal nuclei associated with adverse outcomes (*p* = 0.002). Sensitivity, specificity, PPV, and NPV of later MRI for abnormal outcomes were respectively 82%, 93%, 60%, and 100%.
Spring in ‘t Veld, 2016 [20]	1.5 or 3.0 T; T1, T2	Biometrics: 1D and 2D measurements	Smaller cerebellar width (*p* = 0.025) on 3-month MRI and smaller difference in BG width (*p* = 0.014), thalamic width (*p* = 0.012), and BG surface (*p* = 0.028) between neonatal and 3-month MRI associated with composite adverse outcomes at ≥18 months.
Mulkey, 2012 [21]	1.5 T; T1	Biometrics: volumetrics of whole brain and corpus callosum	Infants with whole brain volumes < 99% CI boundary of the volume estimation model on later MRI had an OR of 33 to have epilepsy, CP, and neurodevelopmental delay (95% CI 2.32–469.03, *p* = 0.008); infants with whole brain volumes > 99% CI boundary of the volume estimation model had an OR of 15 to have a normal outcome (95% CI 1.21–185.46, *p* = 0.029).
Belet, 2004 [22]	Field strength NA; T1, T2, PD	Qualitative: categorization by pattern of injury	Four-month MRI associated with epilepsy (*p* = 0.033), CP (*p* < 0.001), and composite adverse outcomes (*p* < 0.001). Sensitivity, specificity, PPV, and NPV for neurological outcomes were, respectively, 86.7%, 100%, 100%, and 81.8%. *
Tekgul, 2003 [23]	1.5 T; T1, T2	Qualitative: categorization by pattern of injury	Infants with only focal cortical involvement (*p* = 0.045) or myelination delay (*p* = 0.019) more frequently had a favorable outcome compared with the other injury patterns. Infants with only myelination delay were less likely to demonstrate a motor deficit (*p* = 0.016). *
Kragelöh-Mann, 2002 [16]	0.5 or 1.5 T; T1, T2, TSE, FLAIR (subset)	Qualitative: classification of degree of DGM injury	Overall group: severity of DGM injury on MRI ≥ 12 months correlated with motor (*p* = 0.001) and cognitive development (*p* < 0.001). Subgroup with asphyxia and MRI at 2–18 months (*n* = 3): an infant with mild DGM injury had mild motor and cognitive impairment at 15 months. Two infants with severe DGM injury had severe motor and cognitive impairment at, respectively, 27 and 99 months of age.
Byrne, 1990 [25]	1.5 T; T1, T2, IR	Qualitative: assessment of ventricular size, myelination, structural abnormalities, and extracerebral space	Four-month MRI abnormalities were not significantly associated with CP (*p* = 0.580); 8-month MRI abnormalities were significantly associated with CP: PPV of 80%, NPV of 100%, sensitivity of 100%, and specificity of 67%, *p* = 0.015. *
Millet, 1998 [18]	1.0 T; T1, T2, PD	Qualitative: assessment of myelination, corpus callosum size, ventricular and subarachnoid space sizes, and morphology of WM/cortex	Subgroup of term infants with perinatal asphyxia (*n* = 15): diffuse brain injury on later MRI associated with composite abnormal outcomes: PPV of 100%, NPV of 90%, sensitivity of 83%, and specificity of 100%, *p* = 0.002. *
Rutherford, 1996 [26]	1.0 T; T1, T2, IR, FLAIR (subset)	Qualitative: adapted scoring system for later MRI with separate DGM injury score	Significant correlation between the optimality score for neurological function and the injury score for later MRI (*p* = 0.0012) and the separate DGM injury score for later MRI (*p* = 0.0002).
Fujii, 1993 [17]	0.35 or 1.5 T; T1, T2	Qualitative: assessment of myelination	Overall group: infants with an abnormal outcome (*n* = 11) more often showed delayed myelination on MRI ≥ 2 months (*n* = 9, 82%) than infants with a normal outcome (2/13, 15%), *p* = 0.001. A subgroup analysis for term infants with perinatal asphyxia was not possible. *
Steinlin, 1991 [24]	2.35 T; T1, T2	Qualitative: assessment of gross morphology, myelination, bleeding, and ventricular size	Diffuse MRI lesions on later MRI were not significantly associated with severe motor abnormalities (*p* = 0.070) or the composite of mild and severe motor abnormalities (*p* = 0.192) at follow-up. Numbers were too small to analyze MRI in relation to epilepsy or audiovisual impairment. *

**Table 3:** The main findings of the studies reporting both neonatal MRI and later MRI. BG: basal ganglia; BGT: basal ganglia and thalami; CP; cerebral palsy; DGM: deep gray matter; DWI: diffusion-weighted imaging; FLAIR: fluid attenuated inversion recovery ^1^H-MRS: magnetic resonance spectroscopy; IR: inversion recovery; NPV: negative predictive value; OR: odds ratio; PPV: positive predictive value.

Author, Year of Publication	Neonatal MRI Protocol	Age Neonatal MRI	Association between Neonatal MRI Findings and Neurodevelopmental Outcomes	Neonatal versus Later MRI
Parmentier, 2023 [10]	T1, T2, DWI, ^1^H-MRS of BGT, SWI (subset)	<14 days	Biometrics: neonatal MRI not associated with outcome.	Neonatal MRI had better sensitivity and NPV; 3-month MRI had better specificity and PPV.
Spring in ‘t Veld, 2016 [20]	T1, T2	≤7 days	Qualitative score: total neonatal injury score and neonatal DGM injury sub-score associated with 18–24 month outcomes. Sensitivity, specificity, PPV, and NPV were 67%, 93%, 83%, and 84% for the neonatal DGM sub-score. Infants with adverse 5.5-year outcomes had higher neonatal total (*p* = 0.045) and DGM sub-scores (*p* = 0.002) and lower cerebellum injury scores (*p* = 0.003).	Both neonatal and 3-month MRI biometrics were associated with outcomes.
Mulkey, 2012 [21]	T1, DWI	≤7 days	Infants with an adverse outcome had larger BG and thalamic width (both *p* < 0.001) and BG surface area (*p* = 0.007) on neonatal MRIs compared with infants with a favorable outcome. The cerebellar width on neonatal MRIs was larger in the favorable outcome group (*p* = 0.028).	Both neonatal and later MRI findings were associated with outcomes.
Belet, 2004 [22]	T1, T2	5–19 days	BG injury on neonatal MRI associated with CP (*p* = 0.019) and the presence of CP, epilepsy, and delayed neurodevelopment (*p* = 0.027). Acute brain injury volume in the corpus callosum was associated with epilepsy (OR 24.1, *p* < 0.030). Whole brain acute injury volume was not associated with unfavorable neurodevelopmental outcomes.	Neonatal MRI had better sensitivity and NPV; 4-month and 4-year MRI had better specificity and PPV.
Byrne, 1990 [25]	T1, T2, IR	Not further specified	No significant association between abnormal neonatal MRI (*n* = 3 infants) and outcome was demonstrated.	Only 8-month MRI was associated with outcomes.
Rutherford, 1996 [26]	T1, T2, IR, FLAIR (subset)	≤4 weeks	Significant correlation between the optimality score for neurological function and the neonatal DGM injury score (*p* = 0.0004) and the neonatal MRI injury score (*p* = 0.0034). There was a significant correlation between the neonatal and later DGM injury scores (*p* = 0.0005) and MRI injury scores (*p* = 0.0001).	Both neonatal and later MRI findings were associated with outcomes.
Fujii, 1993 [17]	T1, T2	<2 months	No significant association between MRI findings <2 months and neurologic outcome among overall study population (no subgroup analysis for term infants with perinatal asphyxia).	Only myelination on later MRI was correlated with neurologic outcomes.
Steinlin, 1991 [24]	T1, T2	≤4 days (early) 2–4 weeks (intermediate)	No significant association between abnormal early (*n* = 6 infants) or intermediate MRI (*n* = 11 infants) and outcome was demonstrated.	For the overall group with later MRI at 2–75 months, only later MRI was associated with CP (*p* = 0.031). Neonatal, intermediate, and later MRI at 2–24 months were not associated with outcomes.

## Data Availability

Not applicable.

## Supplementary Materials

The following supporting information can be downloaded at: https://www.mdpi.com/article/10.3390/jcm12247526/s1, Supplement S1: adjusted version of the quality in prognosis studies (QUIPS) review form.

## Appendix A. Search Strategy

### Appendix A.1. PubMed

Items describing infants with NE following asphyxia, later MRI, and neurodevelopmental outcome were searched with free text and Medical Subject Heading (MeSH) search terms as follows:

(Brain Hypoxia-Ischemia*[Title/Abstract] OR Brain Ischemia Hypoxia[Title/Abstract] OR Brain Hypoxia-Ischaemia*[Title/Abstract] OR Brain Ischaemia Hypoxia[Title/Abstract] OR Ischemic-Hypoxic Encephalopath*[Title/Abstract] OR Cerebral Hypoxia Ischemia*[Title/Abstract] OR Cerebral Hypoxia Ischaemia*[Title/Abstract] OR Cerebral Ischemia Hypoxia*[Title/Abstract] OR Cerebral Ischaemia Hypoxia*[Title/Abstract] OR Brain Anoxia-Ischemia*[Title/Abstract] OR Brain Ischemia Anoxia*[Title/Abstract] OR Cerebral Ischemia Anoxia*[Title/Abstract] OR “Asphyxia Neonatorum”[Mesh] OR “Asphyxia”[MeSH Terms] OR asphyx*[Title/Abstract] OR “hypoxia ischemia, brain”[MeSH Terms] OR hypoxic-ischemic encephalopathy[Title/Abstract] OR hypoxic-ischaemic encephalopathy[Title/Abstract]) AND (“Magnetic Resonance Imaging”[MeSH Terms] OR “magnetic resonance imag*”[Title/Abstract] OR MRI[Title/Abstract]) AND (“Infant, Newborn”[Mesh] OR “Infant”[MeSH Terms] OR infant*[Title/Abstract] OR newborn*[Title/Abstract] OR baby[Title/Abstract] OR neonat*[Title/Abstract] OR babies[Title/Abstract]) AND (Serial* OR follow-up OR repeat* OR late mri OR evolution* OR transformation* OR evolv*)

### Appendix A.2. Embase

Items describing infants with NE following asphyxia, later MRI, and neurodevelopmental outcome were searched with free text and Emtree terms as follows:

(‘Infant’/exp OR ‘infant*’:ti,ab,kw OR ‘newborn*’:ti,ab,kw OR ‘baby’:ti,ab,kw OR ‘neonat*’:ti,ab,kw OR ‘babies’:ti,ab,kw) AND (‘Brain Hypoxia-Ischemia*’:ti,ab,kw OR ‘Brain Ischemia Hypoxia’:ti,ab,kw OR ‘Brain Hypoxia-Ischaemia*’:ti,ab,kw OR ‘Brain Ischaemia Hypoxia’:ti,ab,kw OR ‘Ischemic-Hypoxic Encephalopath*’:ti,ab,kw OR ‘Cerebral Hypoxia Ischemia*’:ti,ab,kw OR ‘Cerebral Hypoxia Ischaemia*’:ti,ab,kw OR ‘Cerebral Ischemia Hypoxia*’:ti,ab,kw OR ‘Cerebral Ischaemia Hypoxia*’:ti,ab,kw OR ‘Brain Anoxia-Ischemia*’:ti,ab,kw OR ‘Brain Ischemia Anoxia*’:ti,ab,kw OR ‘Cerebral Ischemia Anoxia*’:ti,ab,kw OR ‘asphyxia’/exp OR ‘asphyx*’:ti,ab,kw OR ‘hypoxic-ischemic encephalopathy’:ti,ab,kw OR ‘hypoxic-ischaemic encephalopathy’:ti,ab,kw OR ‘hypoxic ischaemic’:ti,ab,kw OR ‘hypoxic ischemic’:ti,ab,kw) AND (‘nuclear magnetic resonance’/exp OR ‘magnetic resonance imag*’:ti,ab,kw OR ‘MRI’:ti,ab,kw) AND (‘serial*’:ti,ab,kw OR ‘follow-up’:ti,ab,kw OR ‘repeat*’:ti,ab,kw OR ‘late MRI’:ti,ab,kw OR ‘evolution*’:ti,ab,kw OR ‘transformation*’:ti,ab,kw OR ‘evolv*’:ti,ab,kw)

## Appendix B

**Table A1:** Results from the risk of bias assessment.

	1	2	3	4	5	6	Overall Quality
Parmentier, 2023 [10]	LR	LR	LR	MR	LR	LR	High
Jose, 2013 [19]	MR	LR	LR	MR	MR	LR	High
Spring in ‘t Veld, 2016 [20]	MR	LR	LR	MR	HR	LR	Moderate
Mulkey, 2012 [21]	MR	LR	LR	HR	HR	MR	Moderate
Belet, 2004 [22]	MR	LR	LR	LR	HR	LR	Moderate
Tekgul. 2003 [23]	MR	LR	LR	MR	HR	HR	Moderate
Krägeloh-Mann, 2002 [16]	MR	LR	LR	MR	HR	HR	Moderate
Byrne, 1990 [25]	MR	LR	MR	MR	HR	HR	Moderate
Millet, 1998 [18]	HR	LR	LR	MR	HR	HR	Low
Fujii, 1993 [17]	HR	LR	LR	HR	HR	MR	Low
Rutherford, 1996 [26]	HR	LR	LR	MR	HR	HR	Low
Steinlin, 1991 [24]	MR	MR	MR	HR	HR	HR	Low
