# Supplementary material for: Cranial MRI beyond the Neonatal Period and Neurodevelopmental Outcomes in Neonatal Encephalopathy Due to Perinatal Asphyxia: A Systematic Review
Source: J Clin Med. 2023 Dec 6;12(24):7526. doi: 10.3390/jcm12247526 (PMC10743759; doi:10.3390/jcm12247526)
Supplement: Supplementary file 1 [file jcm-12-07526-s001.zip › jcm-2734246-supplementary.pdf]

<table>
<tr><td>Author and year of publication</td><td colspan="4"></td></tr>
<tr><td>Reviewer</td><td colspan="4"></td></tr>
<tr><td colspan="5"></td></tr>
<tr><td>Biases</td><td>Issues to consider for judging overall rating of "Risk of bias"</td><td>Study Methods & Comments</td><td>Rating of reporting</td><td>Rating of "Risk of bias"</td></tr>
<tr><td>1. Study Participation</td><td>Goal: To judge the risk of selection bias (likelihood that relationship between late MRI and neurodevelopmental outcome is different for participants and eligible non-participants).

The domain was graded as “low risk” when ≤3 items were graded as medium risk and the remaining items were graded as low risk. The domain was graded as “high risk” when there were ≥3 items graded as high risk. Otherwise, the domain was graded as “medium risk”.</td><td></td><td></td><td></td></tr>
</table>

Source of target population	The source population or population of interest is adequately described.			
Method used to identify population	The sampling frame and recruitment are adequately described, including methods to identify the sample sufficient to limit potential bias			
Recruitment period	Period of recruitment is adequately described			
Place of recruitment	Place of recruitment (setting and geographic location) are adequately described			
Inclusion and exclusion criteria	Inclusion and exclusion criteria are adequately described (e.g. congenital malformations, central nervous system infections).			
Adequate study participation	There is adequate participation in the study by eligible individuals			

Baseline characteristics	The baseline study sample is adequately described for sex, birth weight, gestational age, Apgar scores, pH, and grade of encephalopathy. Low risk: no missing baseline characteristics; moderate risk: 1-2 characteristics missing; high risk: >2 missing characteristics)			
**Summary Study participation**	**The study sample represents the population of interest on key characteristics, sufficient to limit potential bias of the observed relationship between repeat MRI and neurodevelopmental outcome.**			
**2. Study Attrition**	**Goal: To judge the risk of attrition bias (likelihood that relationship between PF and outcome are different for completing and non-completing participants).**<br><br>**The domain was graded as “low risk” when ≤2 items**			

	**were graded as medium risk and the remaining items were graded as low risk. The domain was graded as "high risk" when there were ≥2 items graded as high risk. Otherwise, the domain was graded as "medium risk".**			
Proportion of baseline sample available for analysis	Response rate (i.e., proportion of study sample completing the study and providing outcome data) is adequate.			
Attempts to collect information on participants who dropped out	Attempts to collect information on participants who dropped out of the study are described.			
Reasons and potential impact of subjects lost to follow-up	Reasons for loss to follow-up are provided.			
Outcome and prognostic factor information on those lost to follow-up	Participants lost to follow-up are adequately described for sex, gestational age, birth weight, Apgar scores, pH, grade of encephalopathy<br><br>There are no important differences between sex,			

	gestational age, birth weight, Apgar scores, pH, grade of encephalopathy and outcomes in participants who completed the study and those who did not.			
**Study Attrition Summary**	**Loss to follow-up (from baseline sample to study population analyzed) is not associated with key characteristics (i.e., the study data adequately represent the sample) sufficient to limit potential bias to the observed relationship between PF and outcome.**			
**3. Prognostic Factor Measurement**	**Goal: To judge the risk of measurement bias related to how PF was measured (differential measurement of PF related to the level of outcome).**<br><br>**The domain was graded as "low risk" when ≤2 items were graded as medium risk and the remaining items were graded as low risk.**			

	**The domain was graded as “high risk” when there were ≥2 items graded as high risk. Otherwise, the domain was graded as “medium risk”.**			
Definition of the PF	A clear definition or description of late MRI is provided (e.g. time range in which MRI was performed, imaging protocol, imaging system).			
Valid and Reliable Measurement of PF	Method of late MRI analysis is adequately valid and reliable to limit misclassification bias (e.g. use of validated score or pipeline, multiple independent observers, blinding)			
Method and Setting of PF Measurement	The method and setting of analysis of late MRI is the same for all study participants.			
Proportion of data on PF available for analysis	Adequate proportion of the study sample has complete data for late MRI findings (<10% = low risk; 10-20% =			

	medium risk; >20% = high risk)			
**PF Measurement Summary**	**PF is adequately measured in study participants to sufficiently limit potential bias.**			
**4. Outcome Measurement**	**Goal: To judge the risk of bias related to the measurement of outcome (differential measurement of outcome related to the baseline level of PF).**<br><br>**The domain was graded as "low risk" when ≤1 item was graded as medium risk and the remaining items were graded as low risk. The domain was graded as "high risk" when there was ≥1 item graded as high risk. Otherwise, the domain was graded as "medium risk".**			
Definition of the Outcome	A clear definition of neurodevelopmental outcome is provided, including duration of follow-up			

Valid and Reliable Measurement of Outcome	The method of outcome measurement used is adequately valid and reliable to limit misclassification bias			
Method and Setting of Outcome Measurement	The method and setting of outcome measurement is the same for all study participants.			
**Outcome Measurement Summary**	**Outcome of interest is adequately measured in study participants to sufficiently limit potential bias.**			
**5. Study Confounding**	**Goal: To judge the risk of bias due to confounding (i.e. the effect of PF is distorted by another factor that is related to PF and outcome).**<br><br>**The domain was graded as "low risk" when ≤2 items were graded as medium risk and the remaining items were graded as low risk. The domain was graded as**			

	**"high risk" when there were ≥2 items graded as high risk. Otherwise, the domain was graded as "medium risk".**			
Important Confounders Measured	Important potential confounders, including treatment, are measured/reported (e.g. degree of encephalopathy, presence of seizures, PMA at performance of late MRI, hypothermia, drugs)			
Definition of the confounding factor	Clear definitions of the important confounders measured/reported are provided			
Valid and Reliable Measurement of Confounders	Measurement of all important confounders is adequately valid and reliable			
Method and Setting of Confounding Measurement	The method and setting of confounding measurement are the same for all study participants.			
Appropriate Accounting for Confounding	Important potential confounders are accounted for in the study design (e.g.,			

	matching for key variables, stratification, or initial assembly of comparable groups).<br><br>Important potential confounders are accounted for in the analysis (i.e., appropriate adjustment).			
**Study Confounding Summary**	**Important potential confounders are appropriately accounted for, limiting potential bias with respect to the relationship between PF and outcome.**			
**6. Statistical Analysis and Reporting**	**Goal: To judge the risk of bias related to the statistical analysis and presentation of results.**<br><br>**The domain was graded as "low risk" when ≤1 item was graded as medium risk and the remaining items were graded as low risk. The domain was graded as "high risk" when there was ≥1 item graded as high risk.**			

	**Otherwise, the domain was graded as "medium risk".**			
Presentation of analytical strategy	There is sufficient presentation of data to assess the adequacy of the analysis.			
Model development strategy	The selected statistical model is adequate for the design of the study.			
Reporting of results	There is no selective reporting of results.			
